# Submucosal oesophageal squamous cell carcinoma with lymph node metastasis: a case report and literature review

**DOI:** 10.1186/s12876-022-02169-1

**Published:** 2022-03-03

**Authors:** Yuting Jia, Quanmao Zhang, Erfeng Li, Zhen Zhang, Xing Chen

**Affiliations:** 1 Department of Endoscopy Center, Shanxi Cancer Hospital, Taiyuan, China; 2 Department of Pathology, Shanxi Cancer Hospital, Taiyuan, China

**Keywords:** Submucosal oesophageal squamous cell carcinoma, Endoscopic submucosal dissection, Submucosal lesions, Case report

## Abstract

**Background:**

Submucosal oesophageal squamous cell carcinoma is a quite infrequent and special type of oesophageal cancer. Its endoscopic manifestations are similar to those of submucosal oesophageal lesions, so it is easily ignored or misdiagnosed. Thus, the exact and timely diagnosis of oesophageal subepithelial lesions (SELs) is crucial. Endoscopic submucosal dissection (ESD) improves the diagnosis rate of malignant SELs without specific endoscopic presentations.

**Case presentation:**

We report a 63-year-old patient with submucosal lesions of the oesophagus under endoscopy, but CT suggested mediastinal lymphadenectasis. Thus, there was a contradiction between them. After multidisciplinary consultation, endoscopic submucosal dissection (ESD) resection was finally recommended. The lesion was completely resected by endoscopic submucosal dissection. Postoperative pathology reported poorly differentiated squamous cell carcinoma, and subsequent PET-CT examination provided clarity, revealing mediastinal lymph node metastasis.

**Conclusions:**

Not all oesophageal SELs are benign, and a small number of SELs can be malignant. Submucosal oesophageal squamous cell carcinoma is a rare disease that may be characterized by oesophageal subepithelial lesions (SELs). Therefore, the precise and timely diagnosis of SELs is essential. If it is necessary to obtain lesion tissue for a definite diagnosis, ESD with less invasiveness is an excellent choice.

## Background

Submucosal oesophageal lesions, namely, oesophageal subepithelial lesions (SELs), are a type of tumour derived from the mesenchymal tissues of the oesophagus rather than the epithelial layer. Their endoscopic features are similar to those of oesophageal submucosal lesions, such as leiomyomas, melanomas, and lipomas, and they account for less than one percent of all oesophageal tumours [1]. SELs present as submucosal eminence lesions protruding into the lumen of the oesophagus with normal epithelial tissue [2]. It is universally believed that submucosal masses are benign lesions; however, these lesions are a heterogeneous group ranging from benign to malignant. Therefore, it is vital to diagnose SELs in a timely and accurate manner, especially malignant lesions. They have no typical clinical symptoms and are often found by accident [3], so they are easily ignored or misdiagnosed [4]. At present, endoscopic ultrasound (EUS) has become the primary means for diagnosing subepithelial lesions. It can provide important information concerning the layer of the lesion and echogenic characteristics, and endoscopic ultrasound-guided fine needle aspiration (EUS-FNA) can be used to distinguish benign and malignant tumours through tissue acquisition. However, both EUS and EUS-FNA technologies have certain limitations. EUS cannot reliably visualize the muscularis mucosa. Moreover, EUS alone is far from sufficient to diagnose lesions located in the third or fourth layer [5]. In addition, EUS-FNA can increase the risk of tumour capsule destruction, which will result in tumour diffusion. At the same time, the oesophageal mucosa is prone to adhesion after a puncture, which brings difficulties to the later operation [6]. In recent years, advances in endoscopic technology and endoscopic submucosal dissection (ESD) have improved the diagnosis rate of malignant SELs that lack characteristic endoscopic manifestations [7]. We report a case of oesophageal SEL resected by ESD. The pathological report revealed poorly differentiated squamous cell carcinoma.

## Case presentation

**Fig. 1:**
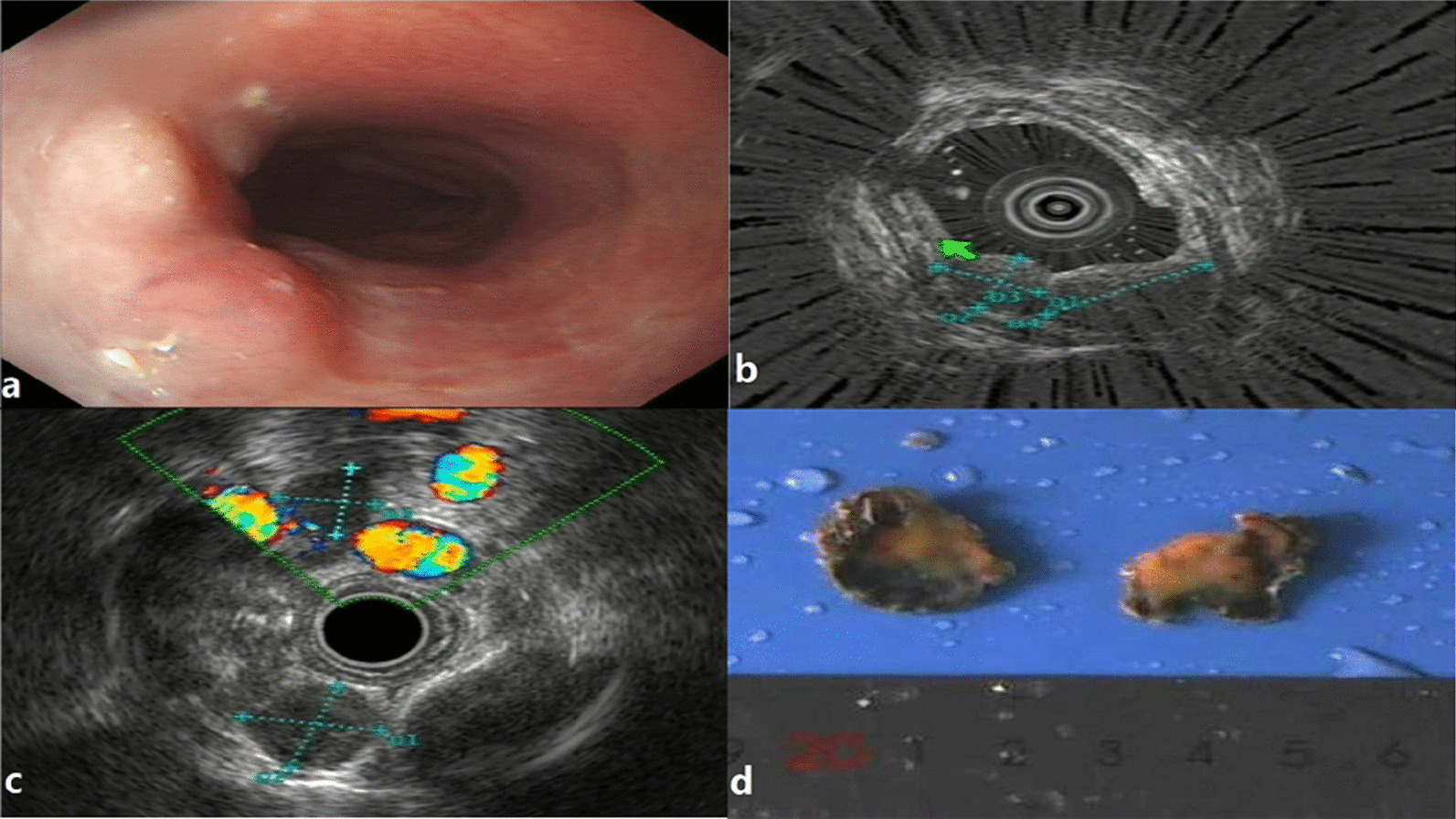
Upper gastrointestinal endoscopy in our case: **a** White light endoscopy shows a 1.5*0.6 cm lesion covered by smooth, normal mucosa; **b** EUS indicates well-defined, slightly heterogeneous, and hypoechoic lesions that might have originated from the submucosal layer; **c** two hypoechoic masses of approximately 1.5*1.5 cm and 1.1*1.0 cm in the mediastinum, with uniform echoes and clear boundaries, which were considered to be enlarged lymph nodes; **d** lesion specimen removed by ESD

**Fig. 2:**
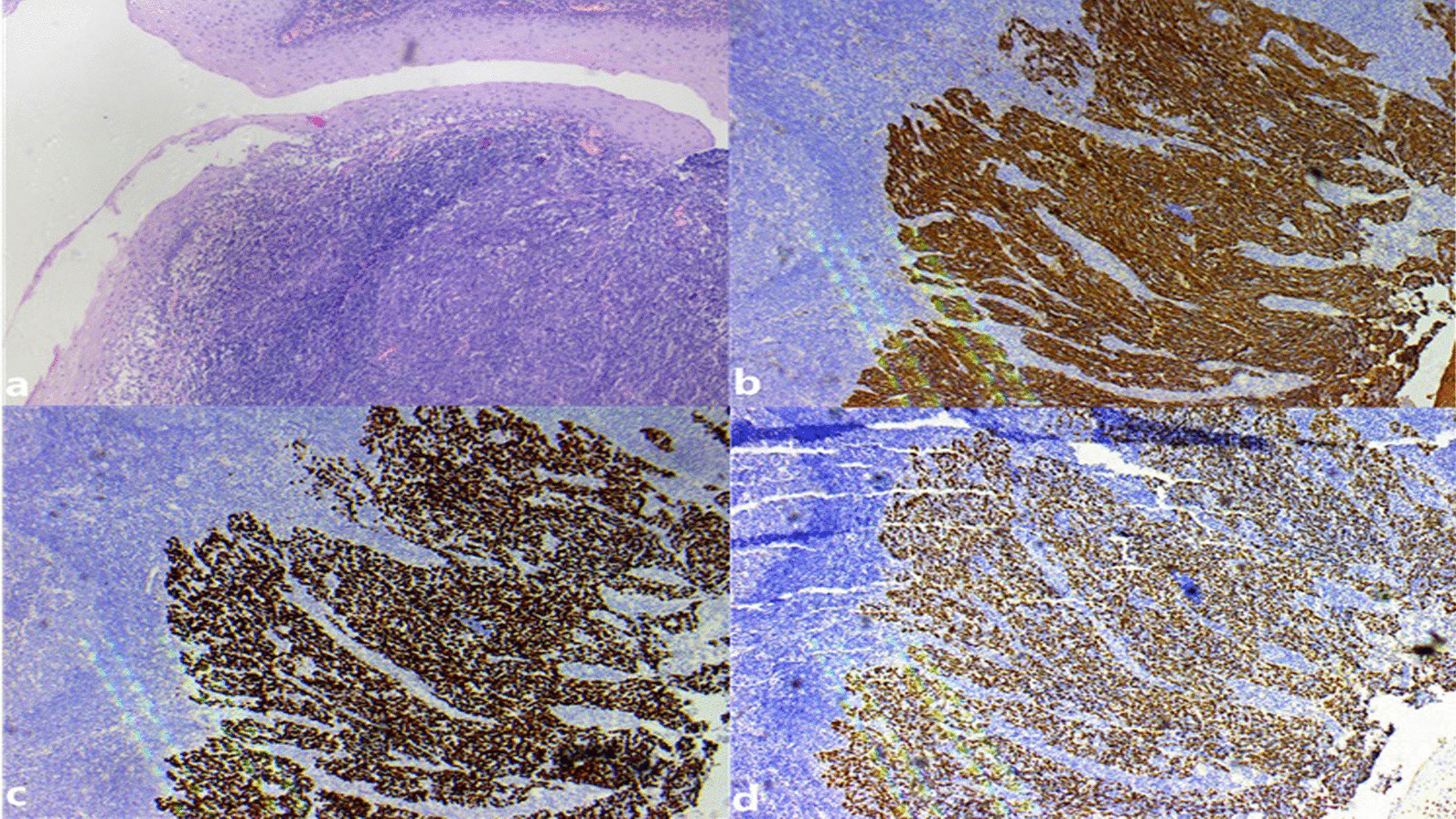
Histopathological examination: **a** heterotypic cells could be seen in the submucosa hyperplastic lymphoid tissue, with nest infiltration and growth. The nuclear atypia is obvious, and the nuclear division is easy to see. Its epithelial layer was intact. However, the classification is still unclear; **b** positive immunohistochemical staining for AE1/AE3, in accordance with epithelial carcinoma; **c** positive immunohistochemical staining for P40, consistent with squamous cell carcinoma; **d** positive immunohistochemical staining for P63, in line with squamous cell carcinoma

**Fig. 3:**
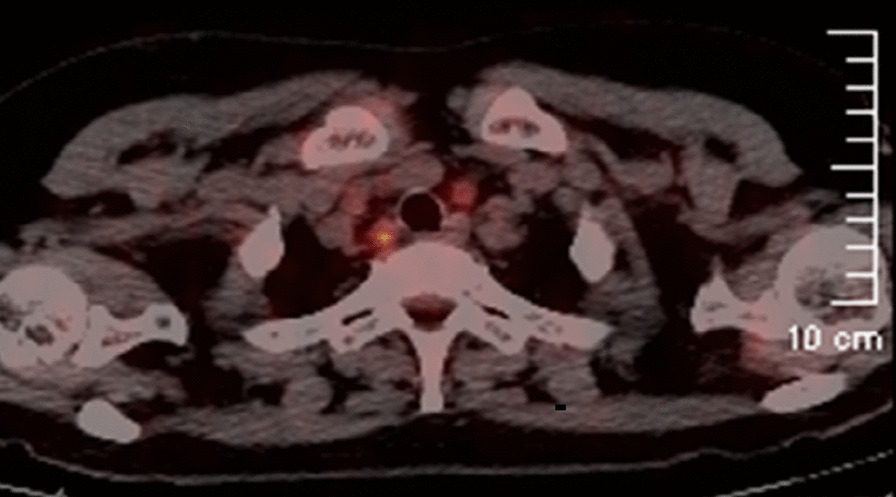
PET scan images showed a hypermetabolic lesion in the bilateral tracheoesophageal groove with an SUVmax of 13.67, which indicated the metastasis of lymph nodes

A 63-year-old patient was admitted to our department on September 14, 2020, due to an protuberant oesophageal lesion, which was found during a physical examination one week prior. She was in good health in the past and had no apparent medical history. The physical examination showed no positive symptoms. Laboratory tests showed no abnormalities in tumour markers. Upper gastrointestinal endoscopy (OLYMPUS GIF-H290) demonstrated a smooth eminence with a normal oesophageal epithelium in the oesophagus 23 cm away from the incisor (Fig. 1a). Endoscopic ultrasound (OLYMPUS GF-UE260) indicated that an apparent bell mouth could be seen in the submucosa, which is a sign of the origin of the disease. This suggested that the lesion might be derived from the submucosal layer (Fig. 1b). Two hypoechoic masses of approximately 1.5*1.5 cm and 1.1*1.0 cm with uniform echoes and clear boundaries could be seen in the mediastinum (Fig. 1c). Chest CT revealed thickening of the oesophageal wall, and small lymph nodes were seen in the bilateral tracheoesophageal groove with a short diameter of approximately 0.8 cm. Combined with white light endoscopy and endoscopic ultrasound, we thought that the lesion might have originated from the submucosa. Therefore, we did not perform an endoscopic biopsy. After multidisciplinary consultation, endoscopic lesion resection (ESD) was recommended to further define the diagnosis and treatment. On October 14, ESD was performed to completely resect the lesion, and the specimen was sent for examination. The pathological results suggested that heterotypic cells were distributed in nests among proliferative lymphoid tissues in the submucosa. The epithelial layer was intact, and there was no involvement of cancer cells. Cancer cells were visible in the submucosa (Fig. 2a). The immunohistochemistry results demonstrated staining for cytokeratin (CK5), AE1/AE3 (Fig. 2b), P40 (Fig. 2c) and P63 (Fig. 2d), and approximately 80% of the cells were positive for Ki67. Among them, AE1/AE3 positivity was consistent with epithelial carcinoma, while positive staining for CK5, P40 and P63 was in line with squamous cell carcinoma. Furthermore, staining for CD3, CD20, CGA, and Syn was negative, which excluded the possibility of neuroendocrine tumours, lymphoma, etc. These abnormal cells were consistent with poorly differentiated squamous cell carcinoma. Then, the PET scan provided clearer results, revealing 0.5–1.6 cm enlarged lymph nodes in the bilateral tracheoesophageal groove and the right side of the main trachea, with hypermetabolism and an SUVmax of 13.67. This result suggested malignant metastasis of the lymph nodes (Fig. 3). The final clinical diagnosis of this patient was submucosal oesophageal squamous cell carcinoma with lymph node metastasis. Then, the patient started chemoradiotherapy. We followed up with the patient regularly. Two months after the start of radiotherapy and chemotherapy, the patient consciously developed nodular masses in the neck. CT examination showed that the enlarged lymph nodes in the bilateral tracheoesophageal groove were significantly reduced. Cervical colour Doppler ultrasound showed that new swollen lymph nodes appeared in the neck, which were considered metastases. The patient continued to receive treatment. At present, nearly 1 year after surgery, chest CT showed that the mediastinal lymph nodes continued to shrink. Neck CT showed no swelling of neck lymph nodes, and there were no obvious abnormalities in tumour markers or abdominal CT. Oesophagography showed that there was no sign of recurrence after surgery.

## Discussion and conclusions

Most oesophageal squamous cell carcinomas are derived from dysplasia of the oesophageal epithelial layer and mainly manifest as polypoidal masses, with roughness or ulcerative growth [8]. In contrast, submucosal oesophageal tumours are a unique group of lesions that originate from the submucosa or muscularis propria. Submucosal oesophageal squamous cell carcinoma is a particular type of oesophageal cancer with a low incidence. To date, only 7 cases have been reported in the literature (Table 1 has been added at the end of the passage). The first case of submucosal oesophageal squamous cell carcinoma was reported by McGregor et al. in 1976. The patient received radiation therapy and bleomycin chemotherapy and eventually died 7 weeks after the last radiotherapy. An autopsy was performed, and the results showed that cancer cells might originate from the squamous epithelium of intramural cysts located in the muscularis and deep submucosa [9]. Von Rahden et al. [10] reported a case of oesophageal squamous cell carcinoma with an intramural growth pattern, which was diagnosed by postoperative pathology. Schmitz et al. [11] described an intramural mucosal squamous cell carcinoma of the gastroesophageal junction. In addition, Sonthalia et al. presented a rare primary intramural SCC, which was diagnosed by EUS-FNA [12]. Kishino et al. reported oesophageal intramucosal squamous cell carcinoma with gastric metastasis characterized by submucosal lesions [13]. Recently, Zhu He et al. [14] reported 2 cases of submucosal lesions resected by endoscopic mucosal dissection, and the postoperative pathology revealed oesophageal intramural squamous cell carcinomas. Among these 7 patients, the main clinical symptom was dysphagia. Some patients had already developed distant metastases when they were diagnosed, and the prognosis was poor.

**Table 1:** Characteristics of patients with submucosal oesophageal squamous cells

No	Age/sex	Clinical presentation	Upper gastrointestinal endoscopy	Endoscopic ultrasound	Size (cm)	metastasis	Pathology	Treatment	Follow-up outcome
1	65/male	Dysphagia, weight loss	Concentric narrowing of the oesophagus	–	4–5	None	Cancer cells originated from squamous epithelial cysts in the oesophageal wall	Chemoradiotherapy	7 weeks Patient died
2	58/male	Dysphagia		No unequivocal findings	3	Resection margin positive	Poorly differentiated squamous cell carcinoma, infiltrating all layers of the oesophageal wall	Surgery + radiochemotherapy	13 months in fine general condition
3	58/male	Dysphagia, weight loss	–	–	7 × 5 × 5	None	Squamous epithelial malignancy lacking glandular formation	Surgery	6 months in fine general condition
4	45/female	Dysphagia, weight loss	Smooth stricture with normal overlying mucosa	Heteroechoic mass lesion in submucosa	3.3 × 2.5	Bone	Squamous cell carcinoma beneath t normal epithelium	Palliative chemoradiotherapy	2 months No change in the size of the tumour
5	60/male	Dysphagia	Small mass in the oesophagus and a large submucosal mass in the gastric cardia	–	–	Gastric	Oesophageal squamous cell carcinoma with submucosal growth pattern	Surgery	110 days Patient died
6	63/male	Abdominal distension	Mucosal eminence with normal overlying mucosa	Heterogeneous and hypoechoic in muscularis mucosa or submucosa	1.3 × 1.0 × 0.3	Vertical margin positive	Under normal epithelium, atypical cells with an increased nuclear-to-cytoplasmic ratio could be seen	ESD + radiotherapy	2 years No recurrence or metastasis
7	65/male	Dysphagia	Hemispherical lesion with smooth mucosa	Hypoechoic and heterogeneous echotexture in the submucosa	1.5 × 1.5 × 1.0	None	Atypical squamous cell clusters could be seen under normal epithelium	ESD	1 year No recurrence and metastasis

The difference between our case and the previous reports is that, under endoscopic ultrasound, the lesion might be derived from the submucosal layer. The possibility of benign lesions was high, but CT suggested mediastinal lymphadenopathy. Therefore, there was a contradiction between them. However, the patient refused to complete CT-guided lymph node biopsy because of economic issues. At the same time, puncture biopsy guided by EUS may cause adhesion of the mucosa and submucosal tissues, which will increase the difficulty of the operation and may cause bleeding, perforation, and other consequences in severe cases. After multidisciplinary consultation, the patient was eventually recommended to undergo ESD surgery. The pathological report indicated poorly differentiated squamous cell carcinoma, but the epithelial layer was intact and not involved. In contrast, cancer cells were visible in the submucosal layer. Subsequently, PET-CT confirmed the metastasis of mediastinal lymph nodes. Meanwhile, PET-CT also excluded the possibility of metastatic cancer from other parts. We report the first case of submucosal oesophageal squamous cell carcinoma with lymph node metastasis diagnosed by ESD. There are two assumptions about the pathogenesis of submucosal oesophageal squamous cell carcinoma [12]. The first hypothesis is that the tumour may originate from oesophageal cysts or squamous epithelium in the diverticula or squamous cell metaplasia of oesophageal glands. First, the glandular epithelium is simply hyperplastic, then squamous epithelial metaplasia, dysplasia, and finally carcinoma in situ occur, followed by invasive carcinoma. The other hypothesis is that the lesion originates from squamous epithelial cells and spreads to the submucosa through ducts. These cells may have the ability to grow and penetrate under the intact epithelium. Later, the patient underwent chemoradiotherapy. Two months after the operation, a re-examination of chest CT showed that the mediastinal lymph nodes were smaller than before. However, cervical colour Doppler ultrasound showed new metastases to the cervical lymph nodes. Some scholars believe that oesophageal cancer in the submucosa is prone to skip metastasis [15], which may be due to the great lymphatic vessels in the submucosa. Therefore, when cancer occurs in the submucosa, lymphatic metastasis can occur even with early microscopic cancers. In our case, the patient’s disease was discovered during the physical examination. She had no symptoms. Therefore, this situation also suggests the importance of endoscopic physical examination.

To date, a total of 8 patients with this type of submucosal oesophageal squamous cell carcinoma have been reported, including the one we reported. Two of the patients received palliative chemoradiotherapy, and one patient eventually died. The other patient showed no significant change in lesion size after two months of follow-up. Two patients underwent ESD surgery, followed by sequential chemoradiotherapy. One patient was followed up for two years, and the general condition of the patient was good. In our case, the patient’s lymph nodes gradually shrank during nearly 1 year of follow-up after ESD surgery. Our case suggested that ESD surgery should be performed as soon as possible for patients with highly suspected submucosal oesophageal squamous cell carcinoma, and postoperative adjuvant chemoradiotherapy should be performed as quickly as possible to improve the clinical prognosis of patients.

The endoscopic manifestations of submucosal oesophageal squamous cell carcinoma are similar to those of benign oesophageal lesions, so it is easy to misdiagnose. For some patients with submucosal lesions, regular follow-up rather than surgical treatment is recommended. For these patients, if it is not detected in time, the optimum treatment time will be missed. Submucosal oesophageal carcinoma with lymph node metastasis is rare, and in this case, endoscopic ultrasound indicated that the lesion might be derived from the submucosal layer. Most oesophageal submucosal masses are benign lesions, which misleads our preliminary clinical diagnosis. Therefore, a timely and accurate clinical diagnosis of SLE is necessary. Endoscopic submucosal dissection is the priority option for any suspected malignant SEL, which allows not only complete dissection but also provides complete pathological data of the affected tissue.

## Limitations

There were some limitations in our case. First, white light endoscopy revealed that the surface of the lesion was smooth, and the colour was the same as that of the surrounding mucosa. At the same time, ultrasound endoscopy suggested that the lesion might be derived from the submucosal layer. Considering both the white light endoscopy and endoscopic ultrasound findings, we considered that the lesion originated from the submucosa, so we did not perform narrow band imaging (NBI) and magnification. At the same time, no pathological biopsy was performed to rule out nonepithelial lesions. However, this was indeed our negligence. Second, because of economic issues, the patient refused to undergo CT-guided lymph node biopsy, which was also a limitation of our case.

## Data Availability

Not applicable.
